# A Novel Design Combining Isothermal Exponential Amplification and Gold-Nanoparticles Visualization for Rapid Detection of miRNAs

**DOI:** 10.3390/ijms19113374

**Published:** 2018-10-28

**Authors:** Jiquan Jiang, Bin Zhang, Chi Zhang, Yifu Guan

**Affiliations:** 1 Department of Biochemistry and Molecular Biology, College of Medical Laboratory and Technology, Harbin Medical University (Daqing), Daqing 163000, China; jiangjiquan55@163.com; 2 Department of Biochemistry and Molecular Biology, China Medical University, Shenyang 110122, China; posseidonz@163.com (B.Z.); 18647066627@163.com (C.Z.)

**Keywords:** miRNAs, exponential amplification reaction (EXPAR), AuNPs, colorimetric method

## Abstract

MicroRNAs (miRNAs) play important roles in a wide range of biological processes, and their aberrant expressions are associated with various diseases. The levels of miRNAs can be useful biomarkers for cellular events or disease diagnosis; thus, sensitive and selective detection of microRNAs is of great significance in understanding biological functions of miRNAs, early-phase diagnosis of cancers, and discovery of new targets for drugs. However, traditional approaches for the detection of miRNAs are usually laborious and time-consuming, with a low sensitivity. Here, we develop a simple, rapid, ultrasensitive colorimetric assay based on the combination of isothermal Exponential Amplification Reaction (EXPAR) and AuNP-labeled DNA probes for the detection of miRNAs (taking let-7a as a model analyte). In this assay, the presence of let-7a is converted to the reporter Y through EXPAR under isothermal conditions. The subsequent sandwich hybridization of the reporter Y with the AuNP-labeled DNA probes generates a red-to-purple color change. In other words, if the reporter Y is complementary to the AuNP-labeled DNA probes, the DNA-functionalized AuNPs will be aggregated, resulting in the change of solution color from red to purple/blue, while when the AuNP-labeled DNA probes are mismatched to the reporter Y, the solution remains red. This assay represents a simple, time-saving technique, and its results can be visually detected with the naked eye due to the colorimetric change. The method provides superior sensitivity, with a detection limit of 4.176 aM over a wide range from 1 nM to 1 aM under optimal conditions. The method also shows high selectivity for discriminating even single-nucleotide differences between let-7 miRNA family members. Notably, it is comparable to the most sensitive method reported to date, thus providing a promising alternative to standard approaches for the direct detection of let-7a miRNA. Importantly, through combination with specific templates, different miRNAs can be converted to the same reporter Y, which can hybridize with the same set of AuNP-labeled DNA probes to form sandwich hybrids. The color change of the solution can be observed in the presence of the target miRNA. This technique has potential as a routine method for assessing the levels of miRNAs, not only for let-7, but also for various miRNAs in the early phase of cancers. In addition, it can be a useful tool in biomedical research and clinical diagnosis, as well as diagnosis or surveillance programs in field conditions.

## 1. Introduction

MicroRNA (miRNA) is a class of small (approximately 18–24 nucleotides long), single-stranded and non-coding RNAs [1]. They are derived from endogenous short hairpin transcripts and can be found in a variety of species, including plants, and human and animal tissues [2]. Recently, the biological functions of miRNAs have become an area of intense investigation. To date, over 2000 human miRNAs have been identified, more than 30% of which can regulate the human genome [3,4]. MiRNAs can lead to target degradation or translational repression through incorporation into an active RNA-induced silencing complex (RISC) [5]. In general, miRNA regulates protein synthesis post-transcriptionally by binding to the 3′-UTR of their target messenger RNAs (mRNAs), which further influences biological processes such as cell differentiation, apoptosis, proliferation, development, metabolism, immunological response, tumorigenesis, and viral infection [3]. In particular, increasing evidence has revealed that aberrant expression profiles of certain miRNAs in the tissue or blood samples of patients is closely associated with a variety of diseases and disorders, such as human cancers, cardiovascular diseases, viral infections and autoimmune diseases [2]. Therefore, miRNAs have been regarded as a promising class of potential targets for the discovery of new drugs in disease diagnosis and therapy and prognosis, as well as new biomarkers for many diseases, especially cancers. Despite their increasingly well-understood significance in biological functions, the development of miRNA detection techniques has lagged behind due to the unique characteristics of miRNA [6]: (1) low miRNA abundance in samples, (2) highly homogenous sequences with as few as one base difference among miRNA family members, (3) the intrinsic vulnerability to enzymatic digestion, and (4) other characteristics of miRNA such as the short small size also aggravate the difficulty in miRNA detection [7]. Hence, as a novel class of tumor markers, the development of rapid, sensitive and selective methods for identification and quantitation of miRNA is of great significance in basic research, as well as for clinical applications [8].

To meet the urgent need for miRNA expression analysis, several conventional approaches have been developed to identify and quantify miRNAs over the last decades, including Northern blotting analysis, the microarray-based technique and real-time quantitative polymerase chain reaction (qRT-PCR) [9]. Northern blotting analysis is now considered the “gold standard”. Unfortunately, Northern blotting is time-consuming, low-throughput and usually requires large amounts of samples, which limits its application in clinical diagnosis [1,6]. Microarray is being used more and more for miRNA-expression analysis as a result of its high-throughput screening capability. Nevertheless, this process is unsatisfactory due to its low sensitivity and specificity. In addition, microarray suffers from cross-contamination, which also limits its application in clinical diagnosis. qRT-PCR has the advantages of high sensitivity and accuracy, but the short length of miRNA complicates the design of the primers, especially in some complex clinical samples [10]. Therefore, it is of great urgency and severe necessity to develop a novel method for rapid, low-cost, specific miRNA detection [11].

Many isothermal amplification techniques with the distinct advantages of high sensitivity, good specificity and simplicity have emerged as feasible alternative platforms for measuring the expression levels of miRNAs in recent years [12,13,14,15,16,17,18]. These include rolling circle amplification (RCA) [19], exponential amplification reaction (EXPAR), loop-mediated amplification (LAMP) [20,21], strand displacement amplification (SDA) [22], helicase-dependent amplification reaction (HDA) and hybridization chain reaction (HCR) [23].

Among these methods, the isothermal exponential amplification reaction (EXPAR) has gained great attention in profiling low-abundance miRNA because of its high sensitivity, low cost, good tolerance to the inhibitory components in the clinical samples [24]. As an alternative amplification technique, EXPAR is a nonlinear amplification and was devised by Galas and co-workers in 2003 for the rapid and efficient amplification of short oligonucleotides (called triggers). More importantly, EXPAR has the intrinsic merits of isothermal nature, ultra-high amplification efficiency and rapid amplification kinetics by a combination of polymerase strand extension and single-strand nicking [25]. The reaction can provide 10^6^~10^9^-fold oligonucleotide amplification under isothermal conditions within minutes [26]. EXPAR-based methods for the quantitative detection of miRNAs, including quantum dots and catalytic G-quadruplex DNAzyme, were successively developed in different research groups, but they all have complicated handling procedures and vulnerability to contamination.

Recently, the colorimetric assay has gained increasing attention because of its excellent selectivity, its ability to be easily monitored with the naked eye, and its low cost, without the requirement of expensive and sophisticated instruments. Gold nanoparticles (AuNPs) are used in the colorimetric assay owing to their high extinction coefficients and distance-dependent optical properties [27]. AuNP-based colorimetric assay has been widely applied for the detection of DNA, protein, metal ions, and small molecules, as well as the screening of DNA binders [28]. Mirkin and co-workers used gold nanoparticles functionalized with a high density of thiol-modified oligonucleotides for sensitive nucleic acid detection [29]. This involves two sets of thiol-modified oligonucleotide sequences, modified at the 5′- and 3′-terminus, respectively. Both of them were fixed on the surface of AuNPs through Au–S bonds. When the complementary reporter oligonucleotides (amplification products/target nucleotide) were hybridized with AuNP-modified DNA probes, they form a polymeric network of cross-linked AuNPs that will bring the AuNPs close enough together to result in a color change from red to blue (positive result). On the contrary, the mismatched hybridization of reporter oligonucleotides with AuNPs modified DNA probes prevents AuNP aggregation, and the solution remains red (negative result). The presence or absence of the target oligonucleotide can be detected by the naked eye when the color sufficiently changes [30]. For this reason, AuNPs-modified DNA probes offer the advantages of low cost and direct visual detection, compared to those using fluorescence or radioactivity, and can be used widely as tools to establish next-generation detection platforms, showing great potential in the rapid diagnosis of disease [28].

In this study, we demonstrate a novel method for miRNA detection by combining isothermal exponential amplification reaction (EXPAR) and colorimetric visualization of AuNPs to enable the detection of miRNAs in a simple and convenient manner with minimal requirement for instrumentation. To further reduce the cost and complexity of the experiment, only one set of 5′-terminus thiol-modified oligonucleotides were employed for integration with the AuNPs. The change of the fluorescence signal was collected in the DEAOU-408C thermostatic fluorescence detector (Figure S3). Importantly, it is much cheaper in price than the PCR Instrument and the Microplate Reader. Hence, the approach was examined here to determine whether it would rapidly and efficiently detect miRNAs, and to evaluate whether it would be popular in the medical market.

## 2. Results and Discussion

### 2.1. Principle of EXPAR-AuNP Visual Detection

**Scheme 1:**
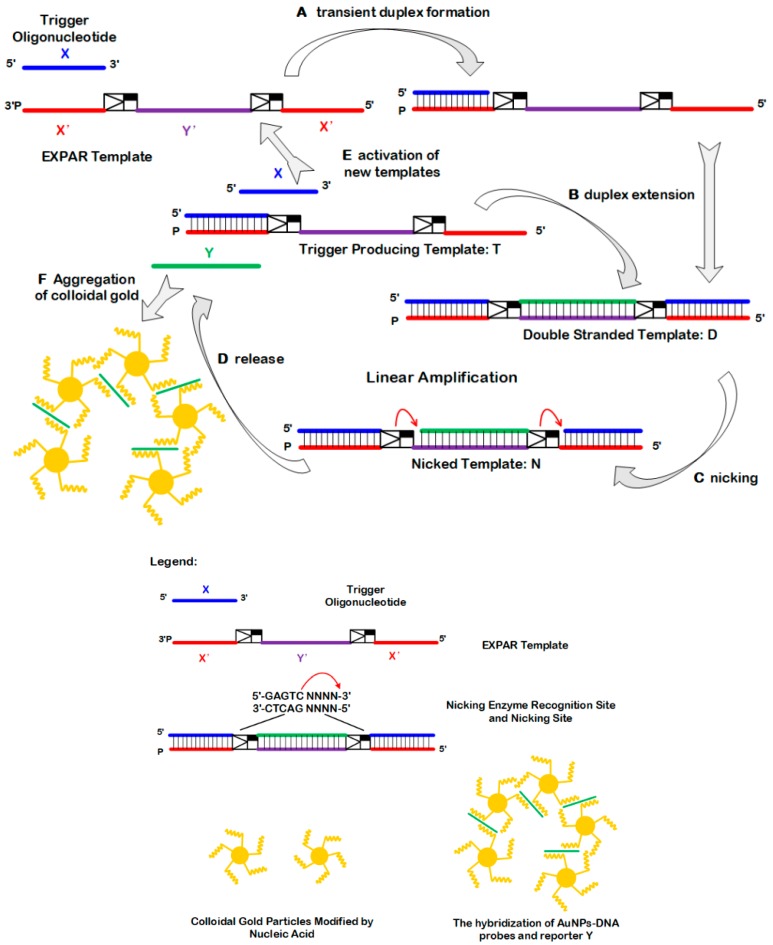
Schematic view of EXPAR-AuNPs analysis of miRNAs. (**A**) Trigger X transiently binds to the complementary sequence at the 3′-end of the amplification template; (**B**) trigger X is extended by DNA polymerase to form a double-strand containing the nickase recognition sequence 5′-GAGTCNNNN-3′ on the top strand; (**C**) at the reaction temperature (55 °C), the top strand is cleaved by nickase Nt.BstNBI; (**D**) the newly formed trigger X and reporter Y were released from the amplification template, the trigger-producing form of the amplification template re-enters the linear amplification cycle, and additional trigger oligonucleotides and reporter Y are generated by double strand extension, cleavage and release; (**E**) the newly formed trigger X activate additional template sequences, giving rise to an exponential amplification of trigger X and reporter Y; (**F**) the exponentially amplified reporter Y will hybridize with the AuNPs-DNA probe, resulting in AuNPs aggregation and significant color changes.

The EXPAR-AuNP visual detection strategy is described in Scheme 1. The EXPAR template X’-Y’-X’ includes three sections: section X’ at the 3′-end (shown in red), section Y’ in the middle (shown in purple), and section X’ at the 5′-end (shown in red). These sections are separated by two identical sequences which are complementary to the nickase recognition site (5′-GAGTC-3′) and cleavage site (four bases downstream), respectively. Here, the sequence of section X’ is complementary to an oligonucleotide called trigger X (shown in blue). Trigger X, corresponding to the short oligonucleotides that initiate EXPAR, can be directly represented by miRNAs or can be indirectly enzymatically generated from specific sites within the target genomic DNA. The detection process contains 6 steps. (A) The trigger X hybridizes transiently with the X’ section at the 3′-end of the EXPAR template; (B) the trigger X is extended on the template by Vent (exo-) polymerase to form a duplex; (C) nickase Nt.BstNBI cleaves the top strand of the duplex to produce single-strand fragments X and Y; (D) fragments X and Y are released from the EXPAR template; (E) the single-strand fragment X is able to hybridize with the other EXPAR templates and act as the prime to initiate EXPAR on additional EXPAR templates. Such cycles could be repeated continuously to create an exponential amplification of single-strand fragment X, as well as single-strand fragment Y; (F) the reporter Y hybridizes with a set of DNA probes (the DNA probes modified with the gold nanoparticles at the 5′-terminus), and such a crosslink reaction causes the aggregation of AuNPs. Consequently, the AuNP solution exhibits color changes which can be visualized directly by the naked eye (Figure S2).

In addition, this new EXPAR-AuNP scheme allows for a flexible experimental design. The sequence of the bridging reporter Y is completely independent from trigger X, so it can be applied to any type of target microRNA. It is only necessary to design a corresponding section X’ of amplification template for specific target sequence, and the Y’ sequence can be used without change. Consequently, generation of the same reporter Y sequence allows specific hybridization with the gold nanoparticle-modified DNA probe. This enables the same set of nanoparticle-modified DNA probes to be used for the detection of any other target microRNAs, eliminating the effort and cost involved in re-synthesizing nanoparticle-modified DNA probes for new experiments to detect different target sequences. Thus, the EXPAR-AuNPs visual detection strategy can be accomplished without requiring any precise and expensive instrument. These features represent important and profound efforts towards the multiplexing assay in the future.

### 2.2. Optimization of EXPAR-AuNP Experimental Conditions

MicroRNA let-7a was chosen as a model for this study owing to the fact that let-7a is abnormally expressed in a wide range of common human cancers, such as lung cancer [3]. To achieve the best detection performance, we investigated the experimental parameters of the EXPAR-AuNP assay by monitoring real-time fluorescence signals (Figure S1).

#### 2.2.1. Reaction Time

**Figure 1:**
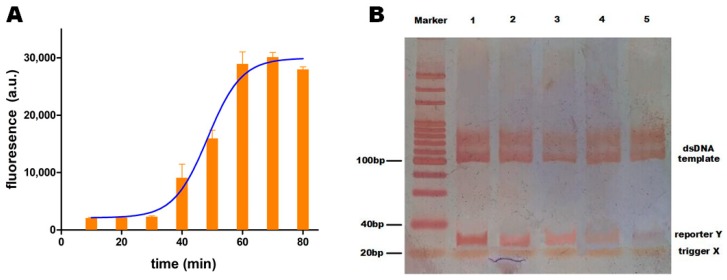
Effect of different reaction periods on EXPAR (Exponential Amplification Reaction). (**A**) EXPAR fluorescence signals monitored by DEAOU real-time fluorescence detector; (**B**) EXPAR results were verified using non-denatured PAGE. The leftmost side of the gel was 20 bp Marker, and lanes 1–5 represent the EXPAR results at 60, 50, 40, 30, and 20 min, respectively.

The influence of the EXPAR amplification time was examined first. It is easy to understand that the longer the reaction time, the greater the amount of reporter Y synthesized. Unfortunately, non-specific amplification reaction would also occur with the time increases, which is a common phenomenon in the isothermal polymerase reaction. As shown in Figure 1A, the fluorescence signal rises very slowly before 30 min, and then increases rapidly from 30 to 60 min, reaching a plateau beyond 60 min, which characterizes the exponential amplification of EXPAR. This EXPAR process was verified using a non-denatured PAGE analysis (Figure 1B). The band of reporter Y (30 bp) became gradually more intensified with the increase of reaction time. Therefore, 60 min was chosen as the optimum EXPAR amplification time in the subsequent experiments.

#### 2.2.2. Reaction Volume and Temperature

**Figure 2:**
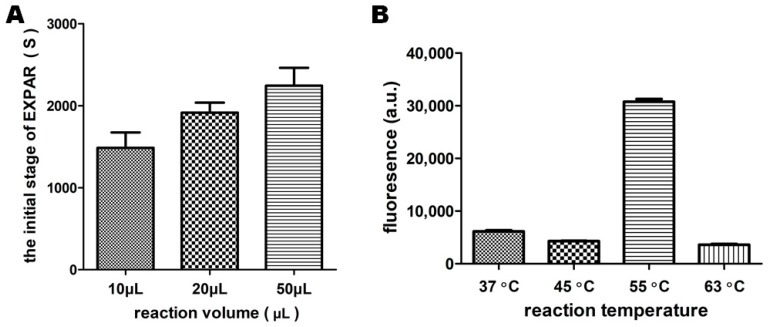
Effect of different reaction volumes and temperatures on EXPAR. (**A**) With the increase in reaction volume, the time of the EXPAR initial stage increased gradually; (**B**) Under different reaction temperatures, experimental results show different fluorescence values.

10, 20 and 50 μL volumes of the sample were prepared for EXPAR, with the EXPAR substrate multiplied accordingly, keeping the concentrations of each component of the EXPAR solution identical. We define the time (in seconds) at which 10% of the maximal fluorescence intensity at the plateau stage as the EXPAR initiation stage. As shown in Figure 2A, with the increase in reaction volume, the time of the EXPAR initial stage increased gradually. In theory, we expect the reaction to enter the initial stage as soon as possible. Obviously, the error bar corresponding to the 20 μL reaction volume was the smallest, indicating that the 20 μL reaction volume showed better repeatability. Therefore, 20 μL was chosen to be the EXPAR total volume for the following experiments. Due to enzyme activity playing a vital role in the efficient performance of EXPAR, and the fact that enzyme activity is highly dependent on temperature, it is extremely important to verify the reaction temperature of EXPAR. As shown in Figure 2B, the EXPAR generates the highest fluorescence signal at 55 °C, and the EXPAR fluorescence signal at other temperatures is only 30% of the highest value at 55 °C. This is probably due to the fact that the nickase Nt.BstNBI is able to achieve its maximal activity at this temperature. Therefore, 55 °C was selected as the working temperature for the subsequent EXPAR experiments.

#### 2.2.3. Fluorescent Dyes and Reaction Template

**Figure 3:**
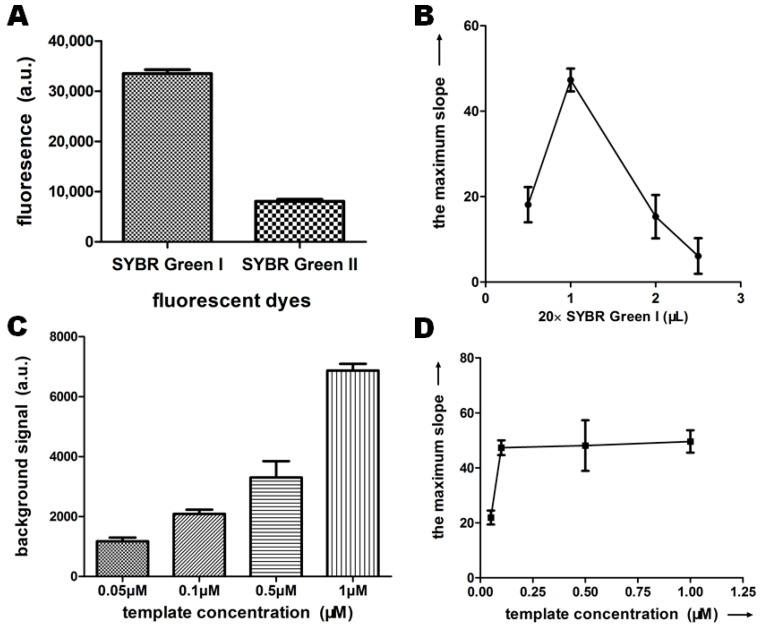
Effect of fluorescent dyes and template quantity on EXPAR. (**A**,**B**) 1 μL of 20× SYBR Green I was adopted as the optimal amount for the subsequent EXPAR; (**C**,**D**) 0.1 μM template was used as the optimum template concentration in 20 μL reaction volume.

The fluorescent dyes SYBR Green I and SYBR Green II were tested as the fluorescence indicators of EXPAR. We examined which one gave rise the highest fluorescence signal when equal amount of fluorescent dyes was used. Figure 3A shows that the fluorescence signal of SYBR Green I is more than three times higher than that of SYBR Green II. Furthermore, as shown in Figure 3B, the results demonstrated that 1 μL of 20× SYBR Green I was able to generate a better fluorescence signal than the others (0.5, 2 and 2.5 μL). Hence, 1 μL of 20× SYBR Green I was adopted as the optimal amount for the subsequent EXPAR. In theory, too little EXPAR template could postpone the initiation of EXPAR, whereas too much EXPAR template could lead to a relatively high fluorescent background, both of which would interfere with the quantitative and sensitive signal readout. To optimize the template concentration, 0.05, 0.1, 0.5 and 1.0 μM of template were used for EXPAR. As shown in Figure 3C, the background fluorescence intensity of EXPAR rises with the increases in template concentration. In particular, when the concentration of the template is 1 μM, the background fluorescence signal is extremely high (above 7000). Moreover, as shown in Figure 3D, the maximum slopes of the EXPAR “S”-curve increase gradually with the increases of the template concentration, and when the template concentration is above 0.1 μM, the increase becomes extremely slow. Considering the factors both of the background signal and the maximum slope of the EXPAR curve, 0.1 μM was used as the optimum template concentration in the following experiments.

#### 2.2.4. Enzymes

**Figure 4:**
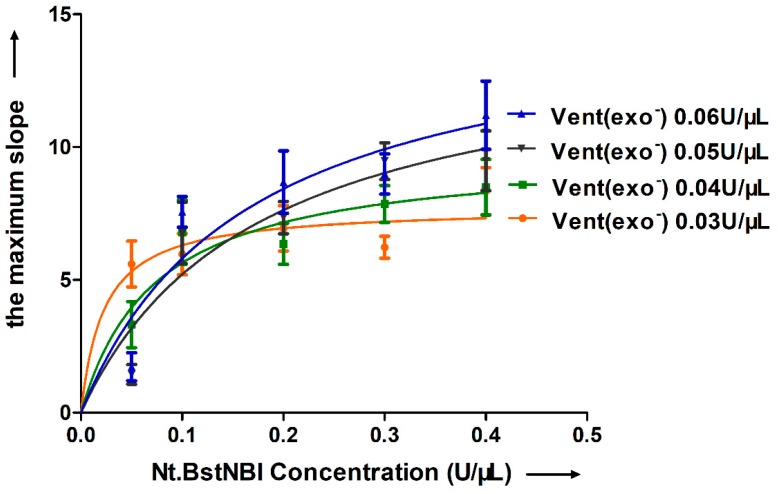
Effect of Vent (exo-) polymerase and Nt.BstNBI nickase on EXPAR. Under the same concentration of one enzyme, the maximal slope of the S-shaped curve increases correspondingly with the concentration of the other enzyme. Considering the cost-effectiveness factor and the excellent experimental performance, 0.06 U/μL polymerase and 0.3 U/μL nickase were selected for the following EXPAR.

It is known that the performance of the EXPAR-AuNPs assay depends heavily upon Vent (exo-) DNA polymerase and Nt.BstNBI nickase. The optimal quantities of Vent (exo-) polymerase and Nt.BstNBI nickase were investigated by measuring the maximal slope of the S-shaped curve of the fluorescence signal. The negative control of each group was treated in the same way without the presence of trigger let-7a. As shown in Figure 4, the maximal slope of the EXPAR S shape-curve gradually increases with the increase of the concentration of nickase under the same polymerase concentration. Meanwhile, under the same concentration of nickase, the maximal slope of the S-shaped curve increases correspondingly with the increase of Vent (exo-) polymerase concentration. However, the change of the maximal slope was considerably slight when the quantities of polymerase and nickase were greater than 0.06 and 0.3 U/μL, respectively. Considering the cost-effectiveness factor and the excellent experimental performance, 0.06 U/μL polymerase and 0.3 U/μL nickase were selected for the following EXPAR in a 20 μL reaction volume.

### 2.3. Evaluation of EXPAR-AuNPs Visual Detection

#### 2.3.1. Sensitivity of the EXPAR-AuNP Assay

**Figure 5:**
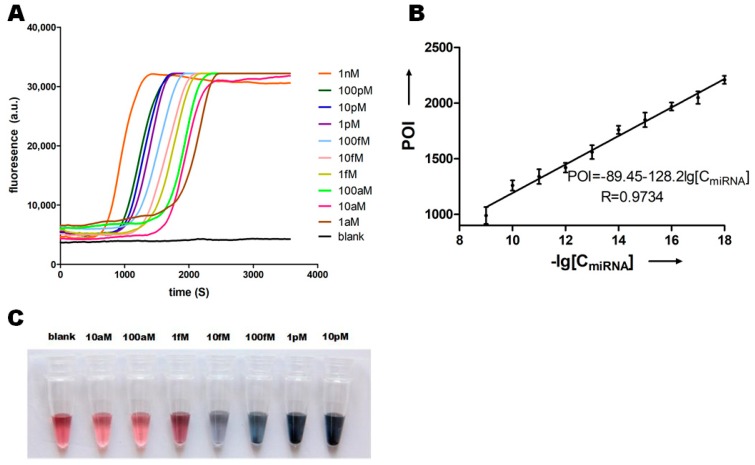
The sensitivity of EXPAR-AuNP visual detection. (**A**) Real-time EXPAR fluorescence signals of let-7a miRNA at different concentrations; (**B**) calibration line of let-7a concentrations versus the POI values; (**C**) EXPAR-AuNPs visual detection of let-7a miRNA at different concentrations.

It has been reported that the expression levels of human let-7 miRNAs family members have the characteristic of tissue specificity, and let-7 miRNAs may be tumor suppressors, as they are poorly expressed in lung cancers [5]. Therefore, we firstly investigated the detection sensitivity of the proposed EXPAR-AuNPs visual assay with the EXPAR fluorescence signal using let-7a miRNA as the intended target. A series of let-7a samples containing different concentrations was prepared, and then used for sensitivity evaluation of the EXPAR assay. With the decrease in the target miRNA concentrations, the fluorescence curves display a gradual rightward shift (Figure 5A). To obtain a quantitative evaluation of the detection sensitivity for let-7a miRNA, we used the point of inflection (POI). The POI is defined as the time corresponding to the maximal slope in the sigmoidal curve [4]. The determined POI values of each concentration were then plotted in Figure 5B. The POI values exhibit an excellent linear relationship with the logarithm (log) of the let-7a miRNA concentrations in the ranges of 1 aM to 1 nM. The correlation equation is POI = −89.45 − 128.2 lg [C_miRNA_] (the correlation coefficient *R* is 0.9734), where [C_miRNA_] is the concentration of let-7a miRNA. For each concentration, the measurements were repeated independently at least three times. Under optimal conditions, a wide dynamic range of more than 9 orders of magnitude was obtained. According to the 3σ method, the limit of detection (LOD) was calculated to be 4.176 aM.

In parallel to the above experiment, we also evaluated the proposed EXPAR-AuNPs visual detection. The EXPAR products of let-7a miRNAs at different concentrations were incubated with the AuNP solution for 10 min, and the color changes were visualized with the naked eye. Figure 5C displays that, with the increase of let-7a concentrations, the color of the AuNPs solution gradually changed from wine-red to blue-violet, and the formation of precipitation can be even noticed, demonstrating a trend consistent with the fluorescence results. In comparison to the previously reported methods, our approach has a much lower LOD. The improvement of sensitivity could be attributed to the high amplification efficiency of EXPAR, as well as the well-designed AuNP-labeled DNA probe.

#### 2.3.2. Specificity of the EXPAR-AuNP Assay

It is still a great challenge to correctly distinguish each member of the miRNA family because of their short lengths and high homologies. It has been reported that discriminating the expression of each let-7 is imperative, because of the important role of these miRNAs in the development of cancer [8]. To evaluate the detection specificity of the proposed EXPAR-AuNPs visual detection, we tested members of let-7 miRNAs family (let-7a–let-7g and let-7i) using the let-7a specific template under identical experimental conditions, as well as a sample without target as a blank control.

**Figure 6:**
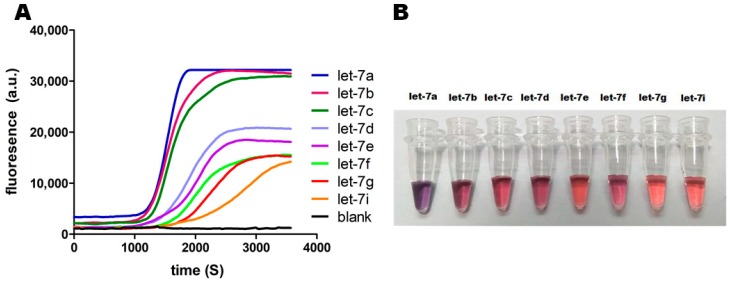
The specificity of EXPAR-AuNPs visual detection. (**A**) Real-time EXPAR fluorescence signals of different let-7 miRNA family members; (**B**) EXPAR-AuNPs visual detection of different let-7 miRNA family members.

As previous described, the proposed assay is based on the extension of the miRNA at the 3′-end to initiate the EXPAR. In comparison with the sequence of let-7a (Table 1), the mismatched nucleotides in let-7b, let-7c, let-7g and let-7i are near the 3′-end, which is expected to create a larger influence on the transient duplex formation, and consequently to lead to an effective discrimination. Figure 6A shows that the fluorescence curve of let-7a is on the topmost, and at the POI of the let-7a, extremely low fluorescence signal was observed for let-7b, let-7c, let-7g and let-7i. In contrast, let-7d, let-7e and let-7f have mismatched nucleotides distant from the 3′-end, and it is expected that the amplified signals produced by let-7d, let-7e and let-7f will be slightly stronger than the signal for the mismatched bases located near their 3′ terminus, and relatively similar to that produced by let-7a. Correspondingly, as shown in Figure 6B, only the EXPAR product for let-7a, which perfectly matches the template, exhibits a color change from wine-red to blue-violet in the colorimetric reaction. Thus, the proposed EXPAR-AuNPs assay with specificity is able to discriminate let-7a from other members of the let-7 miRNA family, even with only a single base mismatch. The good performance at discriminating between the perfectly complementary target and mismatched targets gives this strategy great potential for the analysis of single nucleotide polymorphisms (SNPs).

## 3. Materials and Methods

### 3.1. Materials and Chemicals

All DNA oligonucleotides (HPLC-grade) were synthesized by Sangon Biological Engineering Technology & Services Co., Ltd. (Shanghai, China). HPLC-purity miRNAs, dNTPs mixture and DNA ladder marker were purchased from TaKaRa Biotechnology Co. Ltd. (Dalian, China). The sequences of all DNA oligonucleotides and miRNAs used in this study are given in Table 1. The nickase Nt.BstNBI and Vent (exo-) DNA polymerase, 10× NEBuffer 3.1 and 10× ThermoPol Reaction Buffer were purchased from New England Biolabs (NEB, Beverly, MA, USA). SYBR Green I was purchased from Invitrogen Biotechnology Co. Ltd. (Waltham, MA, USA). Diethylpyrocarbonate (DEPC)-treated water, and Recombinant RNase Inhibitor were purchased from TaKaRa Biotechnology Co. Ltd. All other chemicals (at least analytical grade) were obtained from Sigma-Aldrich (St. Louis, MO, USA). All DNA and miRNAs oligonucleotides sequences were diluted in 1× TE buffer (pH 8.0) to give stock solutions of 100 and 20 µM, respectively. To create and maintain an RNase-free environment, the tips and tubes are RNase-free and require pretreatment to inactivate RNase. All EXPAR solutions were prepared in DEPC-treated deionized water.

### 3.2. Apparatus

All fluorescence measurements of EXPAR were performed on DEAOU-408C thermostatic fluorescence detector (Guangzhou, China). The excitation and detection wavelengths were 470 nm and 520 nm, respectively. The absorption spectra (200–800 nm.) of samples were carried out on UV-Visible Spectrophotometer (Varian Cary 100, Agilent Technologies, Santa Clara, CA, USA).

### 3.3. EXPAR Experiments

The reaction mixtures for EXPAR were prepared separately on ice as Part A and Part B. Part A consisted of 0.5× NEBuffer (50 mM NaCl, 25 mM Tris-HCl (pH 7.9), 5 mM MgCl_2_, and 50 µg/mL BSA (pH 7.9)), template 0.2 µM, target miRNA, RNase Inhibitor 0.8 U/µL and dNTPs 250 µM. Part B consisted of 1× ThermoPol Reaction Buffer (10 mM KCl, 10 mM (NH_4_)_2_SO_4_, 20 mM Tris-HCl (pH 8.8), 2 mM MgSO_4_ and 0.1% Triton-X-100), nickase Nt.BstNBI 0.3 U/µL, Vent (exo-) DNA polymerase 0.06 U/µL, 20× SYBR Green I 1 µL and DEPC-treated water. Part A was heated at 95 °C for 3 min to denature the template and miRNAs, and then cool down on ice. Then 10 µL Part A and 10 µL Part B (in a total volume of 20 µL) were mixed immediately before the fluorescence measurement on DEAOU-408C. The EXPAR reaction was performed at 55 °C for 60 min, temperature fluctuation was controlled within 1 °C. The fluorescence signal was recorded at intervals of 30 s. The EXPAR products quenched and stored at 4 °C for subsequent analysis.

### 3.4. Synthesis of Gold Nanoparticles

Gold nanoparticles (AuNPs) with a diameter of ~13 nm were synthesized by following the reported method of the reduction of hydrogen tetrachloroaurate (HAuCl_4_) with sodium citrate tribasic dihydrate. In brief, 0.5 mL (100 mM) hydrogen tetrachloroaurate was added to DEPC water (45 mL), vigorously stirred and heated to boiling, followed by the addition of 5 mL (38.8 mM) sodium citrate tribasic dihydrate. In 5 min, the solution color changed from light yellow, colorless and dark blue to ruby red, indicating the formation of AuNPs. After 30 min, the heating and stirring were stopped, and then the solution was cooled down to room temperature. The average diameter of gold nanoparticles was verified by transmission electron microscope (TEM) (JEM-100CXII, Tokyo, Japan).

### 3.5. Immobilization of DNA Probes on AuNPs

DNA immobilization on AuNPs was performed according to the procedure reported previously. First, the AuNPs solution (200 µL) was centrifuged at 10,000 rpm for 10 min to remove the excess material, and the supernatant was discarded and the AuNPs precipitate was re-suspended in 200 µL DEPC water. Subsequently, 8 µL ssDNA probe (100 µM) was added and incubated for 12–16 h. Followed by the addition of 5 µL of 2 M NaCl every 12 h. After 3 consecutive days, the final concentration of NaCl was adjusted to 0.3 M. After overnight incubation, the AuNPs labelled DNA probe was centrifuged at 10,000 rpm for 10 min and then the supernatant was discarded. The ruby red precipitates were washed three times with 200 µL phosphate buffer (0.01 M, pH 7.0, 0.3 M NaCl), and then redispersed in the same buffer solution. The DNA-AuNPs was measured by UV–visible spectrophotometer and stored at 4 °C.

### 3.6. AuNP-Based Colorimetric Assay

The AuNP-based colorimetric assay was carried out 10 min by mixing 20 µL EXPAR products with 20 µL DNA probe solution. The subsequent sandwich hybridization generates a red-to-purple color change, allowing the visual detection of miRNAs with the naked eye.

### 3.7. Non-Denatured Polyacrylamide Gel Electrophoresis (nd-PAGE)

Gel electrophoresis was used to analyze the EXPAR products. Samples for gel electrophoresis were prepared as follows: 20 µL EXPAR products or 8 µL DNA ladder were mixed with 2 µL of 10× loading buffer. A non-denatured polyacrylamide gel electrophoresis (9%) of the EXPAR products was carried out in 1× TBE (9 mM Tris–HCl, pH 7.9, 9 mM boric acid, 0.2 mM EDTA) at 200 V for 40 min at room temperature. Thereafter, the gel was stained with silver, and the gel images were captured using a digital camera.

## 4. Conclusions

We have developed a novel platform for simple, rapid, ultrasensitive, semi-quantitative and cost-effective detection of miRNAs by integrating the isothermal EXPAR and DNA-functionalized AuNP colorimetric assay. Under optimal conditions, real-time fluorescence can be monitored with as little as 1 aM of miRNA in DEAOU. Due to the high amplification efficiency of EXPAR, the proposed method can sensitively measure let-7a with a limit of detection (LOD) of 4.176 aM. The sensitivity of our assay is improved by suppressing or eliminating the background amplification in the EXPAR and omitting the additional purification step, avoiding contamination from one sample to the next. Consequently, the sensitivity of the EXPAR-AuNPs assay is comparable to most conventional approaches reported to date.

EXPAR-AuNPs possess several unique features: first, the change of fluorescence signal can be easily monitored with a DEAOU thermostatic fluorescence detector. Due to this novel design, it does not require expensive equipment, offering the advantage of cost efficiency. Second, this approach can be conducted under isothermal conditions (55 °C) without complicated instrument operation, which is convenient for clinical popularization. Third, this method can convert the detection of miRNAs to the detection of reporter Y in a relatively short detection time. Reporter Y is an intermediate step in the assay and does not influence the overall sensitivity for detection of trigger X, which was the analyte of interest. Finally, the overall assay can be conducted in 1 h or less, with minimal reagent consumption. Additionally, this method has the significant advantages of simplicity, direct visualization with the naked eye through the aggregation of DNA-functionalized gold nanoparticles, and low cost, without the requirement of expensive and sophisticated instruments.

Although the EXPAR-AuNP visual detection assay has not been applied in real cell experiments and clinical samples, the experimental results demonstrated that this novel method has great potential to become a valuable tool for sensitive, convenient and specific analyses of miRNA in routine clinical diagnosis, screening of anti-tumor drugs, biomedical research and quarantine/surveillance programs.

## Figures and Tables

**Table 1:** Sequences of the oligonucleotides (In EXPAR template, the nucleotides labeled in bold are the complementary sequences to the recognition site and cleavage site of the cleavage enzyme; the italicized nucleotide is a sequence that can produce reporter Y).

	Sequences	
EXPAR template	5′-AACTATACAACCTACTACCTCA**AACAGACTC***CTGCATTGCCGCAGACTGCATTGCCGCAGA***AACAGACTC**AAACTATACAACCTACTACCTCAA-P-3′	The nucleotides of the normal font are the target gene binding sequences; the nucleotides labeled in bold are the complementary sequences to the recognition site and cleavage site of the cleavage enzyme; the italicized nucleotide is a sequence that can produce reporter Y
let-7a	5′-UGAGGUAGUAGGUUGUAUAGUU-3′	The base of the bold marker is a nucleotide that differs from in let-7a
let-7b	5′-UGAGGUAGUAGGUUGU**G**U**G**GUU-3′	
let-7c	5′-UGAGGUAGUAGGUUGUAU**G**GUU-3′	
let-7d	5′-**A**GAGGUAGUAGGUUG**C**AUAGU-3′	
let-7e	5′-UGAGGUAG**G**AGGUUGUAUAGU-3′	
let-7f	5′-UGAGGUAGUAG**A**UUGUAUAGUU-3′	
let-7g	5′-UGAGGUAGUAG**U**UUGUA**C**AGU-3′	
let-7i	5′-UGAGGUAGUAG**U**UUGU**GCU**GUU-3′	
DNA probes labeled on AuNP	Au-SH-(CH_2_)_6_-TTTTTTTGGGGGGAAAAAAA**CTGCATTGCCGCAGA**-3′	Boldly labeled nucleotides are reporter Y binding sequences
Reporter Y	5′-TCTGCGGCAATGCAGTCTGCGGCAATGCAG-3′	Sequences that complementarily bind to AuNPs-DNA probe

## Supplementary Materials

Supplementary materials can be found at http://www.mdpi.com/1422-0067/19/11/3374/s1.

## Abbreviations

AuNPs	gold nanoparticles
EXPAR	exponential amplification reaction
LOD	limit of detection
POI	point of inflection
