# Supplementary material for: A Novel Design Combining Isothermal Exponential Amplification and Gold-Nanoparticles Visualization for Rapid Detection of miRNAs
Source: Int J Mol Sci. 2018 Oct 28;19(11):3374. doi: 10.3390/ijms19113374 (PMC6275053; doi:10.3390/ijms19113374)

Supplementary Materials

Characterization of AuNPs and AuNPs-DNA probes

The synthesized AuNPs were characterized using transmission electron microscope. The synthetic AuNPs exhibited a nearly perfect round shape, a good monodispersity and uniformity, and the average diameter of ~13 nm. AuNPs solution has a maximum absorbance peak at a wavelength of 520 nm (Supplementary Fig. 1). The DNA probe immobilized AuNPs showed a moderate shift of the surface plasmon resonance band from 520 to 525 nm and a characteristic absorption peak of DNA at 260 nm, which is consistent with that reported in the literature. These results indicate that the DNA probes has been successfully immobilized on the AuNPs. This well-functionalized DNA probes solution maintained the red color and exhibited a high stability under a high salt concentration (0.3 M NaCl).

Feasibility of EXPAR-AuNPs reaction

To verify the feasibility of the proposed EXPAR-AuNPs method, four samples have been prepared: the blank control (BC) containing neither the template nor the trigger, the positive control (PC) containing only the reporter Y without the template and the trigger, the negative control (NC) containing only the template without the trigger, the reaction mixture containing both the template and the trigger. All samples were performed in the identical condition (other reaction components were the same.).

As shown in Supplementary Fig. 2A, the positive control (lane 1) and the negative control (lane 2) exhibited a 30 bp band and a 100 bp band, respectively. In contrast, the EXPAR products which containing both template and trigger exhibited three bands at 20 bp, 30 bp and 100 bp, which correspond to the amplified trigger, the EXPAR amplified reporter Y and the template, respectively. Furthermore, Supplementary Fig. 2B showed colorimetric results of the EXPAR. Both the positive control (PC) and the sample 3 (effective EXPAR product) show significant color changes from wine-red to blue-violet. In contrast, the negative control (NC) and blank control (BC) remain wine-red, suggesting no aggregation of AuNPs. The results further confirmed the feasibility of this approach.

Effect of enzyme on EXPAR-AuNPs reaction

In order to evaluate the feasibility of the proposed EXPAR-AuNPs, let-7a was used as a model to verify the dependence of the enzyme in this method. Theoretically, the absence of a polymerase means that there is no the extension of transient primer/ template duplex, the nickase makes no sense to partially complementary duplex without its recognition and cleavage sites. With polymerase only, the extension of the dsDNA equal to the target mRNA in stoichiometry can be ideally obtained. However, the lack of nickase means that no new trigger X or reporter Y is created, which cannot lead to a continuous amplification cycle. As shown in Supplementary Fig. 3A, the amplification signal of EXPAR with both Vent (exo-) polymerase and nickase Nt.BstNBI was significantly higher than the amplification signal only containing either of them. Similarly, as shown in Supplementary Fig. 3B, EXPAR products containing both polymerase and nickase interacts with the AuNPs labeled DNA probe can lead to significantly color changes (from wine red to blue-violet). This means that the exponential amplification proceeds smoothly and generates a large number of reporter Y. In contrast, the reaction with only one of the enzymes cannot initiate EXPAR, thus the solution color is still wine red. Obviously, this strategy is significantly dependent on Vent (exo-) polymerase and Nt.BstNBI nickase, and when either of them is omitted, neither of the significant fluorescence signal and colorimetric reaction can be observed.

Supplementary Figure 1

Characteristic UV-absorption spectra of DNA, AuNPs and DNA-AuNPs conjugates solutions

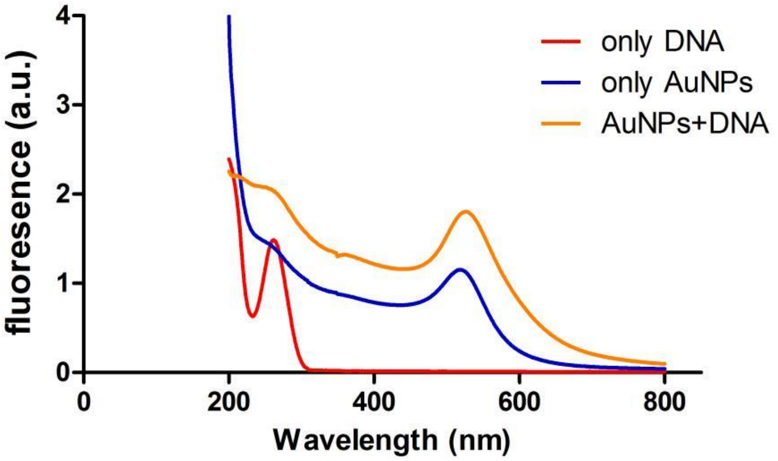

Supplementary Figure 2

Validation of EXPAR-AuNPs product by polyacrylamide gel electrophoresis and colorimetric reaction. A: Non-denatured-PAGE results. The leftmost land is a 20 bp Marker, and lanes 1-3 represent the positive control, the negative control and the reaction mixture, respectively. B: Colorimetric analysis. Tubes 1-4 represent the positive control, the negative control, the reaction mixture and the blank control, respectively

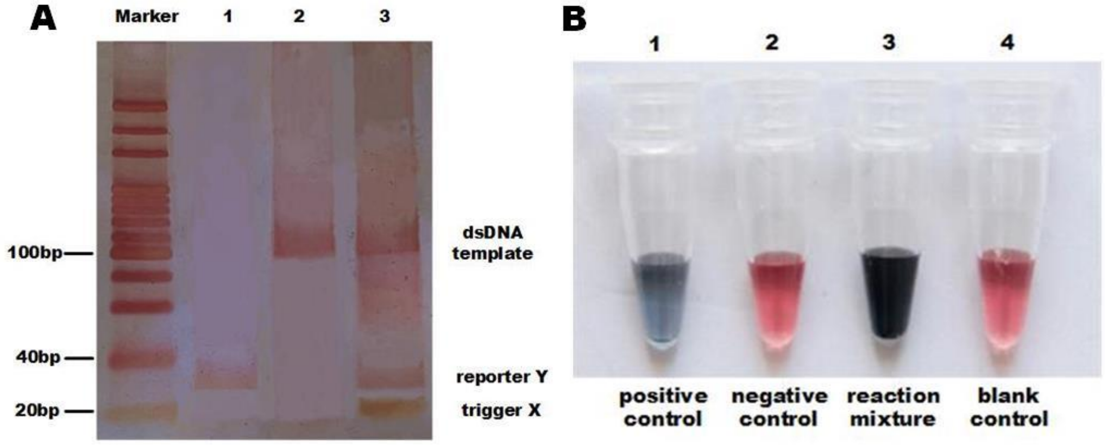

Supplementary Figure 3

Verification of the enzyme dependence. A: EXPAR fluorescence signals monitored by DEAOU real-time fluorescence detector. B: EXPAR products hybridized with AuNPs-labeled DNA probes in colorimetric reaction.

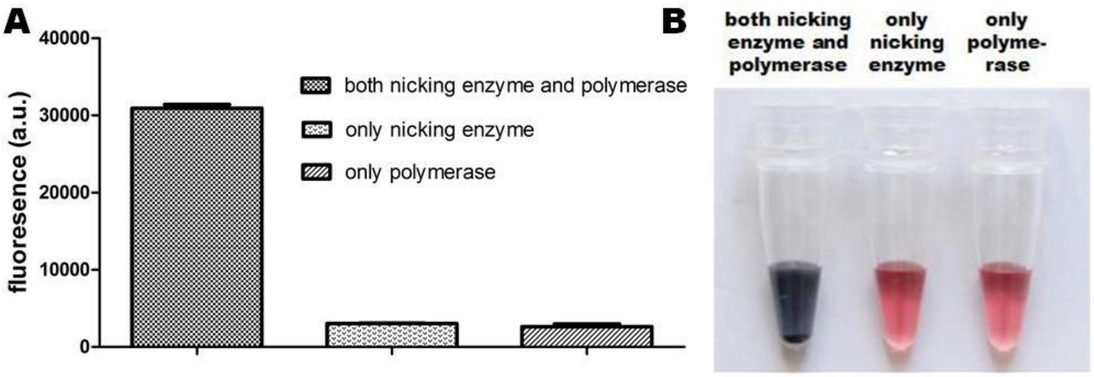

Supplement: Supplementary file 1 [file ijms-19-03374-s001.pdf]
